# Perceived Changes in Communication as an Effect of STN Surgery in Parkinson's Disease: A Qualitative Interview Study

**DOI:** 10.4061/2011/540158

**Published:** 2011-08-14

**Authors:** Emilia Ahlberg, Katja Laakso, Lena Hartelius

**Affiliations:** Division of Speech and Language Pathology, Institute of Neuroscience and Physiology, Sahlgrenska Academy, the University of Gothenburg, 405 30 Göteborg, Sweden

## Abstract

The aim of the present study was to explore four individuals' perspective of the way their speech and communication changed as a result of subthalamic nucleus deep brain stimulation treatment for Parkinson's disease. Interviews of two men and two women were analyzed using qualitative content analysis. Three themes emerged as a result of the analysis. The first theme included sub-themes describing both increased and unexpected communication difficulties such as a more vulnerable speech function, re-emerging stuttering and cognitive difficulties affecting communication. The second theme comprised strategies to improve communication, using different speech techniques and communicative support, as well as trying to achieve changes in medical and stimulation parameters. The third theme included descriptions of mixed feelings surrounding the surgery. Participants described the surgery as an unavoidable dramatic change, associated both with improved quality of life but also uncertainty and lack of information, particularly regarding speech and communication changes. Despite negative effects on speech, the individuals were generally very pleased with the surgical outcome. More information before surgery regarding possible side effects on speech, meeting with a previously treated patient and possibly voice and speech therapy before or after surgery are suggested to facilitate the adjustment to the new speech conditions.

## 1. Introduction

Although subthalamic nucleus deep brain stimulation (STN-DBS) treatment for Parkinson's disease (PD) has been reported to be an effective treatment for advanced motor symptoms of the limbs, such as tremor, rigidity, and bradykinesia, the effects on different speech parameters (phonation, articulation, speech rate) and intelligibility are equivocal [1–3]. Dysarthria was reported as a persistent adverse event in 5–70% of surgical cases reported by Romito and Albanese [2]. A recent study comparing 32 consecutive patients treated with STN-DBS with an optimally medicated control group [3] concluded that most patients exhibited reduced speech intelligibility, a negative change attributed to both medical and surgical factors. Other earlier studies have reported unaffected speech function [4] or improvements [5, 6]. In general, studies of speech effects show that phonatory and articulatory components measured separately are improved by STN-DBS [7–9]. However, speech intelligibility, which is more indicative of overall speech production, is often reduced [3].

The speech disorder associated with PD is well described [10, 11] mainly in terms of perceptually and instrumentally identifiable signs of hypokinetic dysarthria, such as a weak and breathy voice, monotony, imprecise articulation, and variable speech rate. In addition, a few studies include subjective reports of communicative consequences. Miller et al. [12] reported in-depth interviews with 37 individuals. The main concern of these individuals was not the speech and voice changes *per se* but rather their consequences in terms of changed self-concept and restricted participation in social life. These changes were perceived long before changes in speech intelligibility were apparent. Another study [13] administered a self-report questionnaire, the Voice Handicap Index (VHI) [14], to individuals with PD pre- and post-STN-DBS and compared them with a nonsurgically treated group. The VHI scores deteriorated equally in both groups, although, the variability was greater in the surgically treated group. VHI scores and speech intelligibility correlated in both groups, indicating that the individuals' perception of their difficulties was in accordance with an overall measurement of speech deviations.

When comparing studies to evaluate the effects on speech of STN-DBS in individuals with PD, the one consistent finding appears to be variability. This variability may be accounted for by a number of variables: disease-specific variables, type and degree of dysarthria pre- and/or post-surgery, stimulation-related variables, such as location of electrodes, amplitude, and frequency of stimulation, and speech measures chosen and, so on. Small group studies have so far been unable to capture the relevant variables and describe the individuals who might or might not be suitable candidates for surgery. One of the missing perspectives in this area of research appears to be the individual subjective perspective, a perspective that can be expected to contribute to a deeper understanding of the changes in speech and communication as a result of STN-DBS. Conducting qualitative analysis of semistructured interviews is a suitable methodology to explore individual perspectives and describe the heterogeneity of human experiences [15, 16]. Consequently, the aim of the present study was to explore individuals' own perspective of the way speech and communication have changed as a result of STN-DBS.

## 2. Methods

### 2.1. Study Design

Data collection was performed through semi-structured interviews, which were subsequently analyzed using qualitative content analysis [17]. 

### 2.2. Participants

Four individuals were invited to participate in the study. They were selected by the physician in charge of the Motor Disorders Unit at the Neurology Clinic at the local university hospital. The inclusion criteria were Parkinson's disease, at least 2 years after STN-DBS surgery and health status, cognitive and language skills to be able to participate in an interview situation. It was also considered valuable to include both women and men and individuals who had both shorter and longer experience of the effects of STN-DBS. All four were in contact with the Motor Disorders Unit at the time of the study and were selected by the physician as possible and suitable participants. The head of the Neurology Clinic approved the study as a part of the evaluation of surgical treatment in the clinic.

Basic information describing the participants is included in Table 1 (names are pseudonyms). The age range of the 2 women and 2 men was between 61 and 79 years and the time after surgery varied between 2 and 10 years. Years since onset of disease ranged between 10 and 32 years. All participants had had advanced on-off fluctuations for several years before surgery. The participants were assessed by a speech language pathologist (SLP, not involved in the present study) before surgery. Three were considered to have mild to moderate hypokinetic dysarthria and the speech of the fourth was judged to be unaffected. After surgery, assessed 6–12 months after surgery, the participants' dysarthria diagnoses had not changed. One of the four participants had had speech treatment after surgery (Sven). According to medical records regarding cognitive status, Lisa and Anders had no cognitive impairment pre- or post-surgery, Greta had a mild cognitive impairment both pre- and post-surgery and Sven had a mild cognitive impairment after surgery.

### 2.3. Data Collection

Written information regarding the study was sent to the prospective participants, after which they were contacted by telephone. They all agreed to participate and signed a written consent form, including agreeing to the interview being video recorded. They all preferred to be interviewed in their homes. During the interviews, the participants were encouraged to take breaks whenever needed, but no one chose to do so. All the interviews were conducted on a one to one basis, except that the wife of one of the participants was present during the initial part of his interview.

Semistructured qualitative research interviews were conducted and video-recorded by the first author (EA). Prior to actual data collection, two pilot interviews were conducted, with two nonsurgically treated individuals, in order to increase interviewing skills, evaluate the interview guide, and increase trustworthiness. 

A semi-structured interview guide was developed gradually, based on knowledge in the area of research and the pilot interviews. Minor adjustments were made during the course of the four interviews. An interview started with open questions regarding disease history which was followed by more specific questions focusing on experiences of speech and communication after STN surgery. Examples of questions from the interview guide were: “Describe if and how your speech has been affected by DBS treatment?”, “When does your communication work well and when does it not?”, and “How are you able to communicate with other people?—known, unknown?”. The sessions lasted between 45 and 60 minutes. Memos were written in connection with the interviews to obtain a first impression of the content. A second, follow-up interview was conducted by phone with participant number 3 (Anders) to collect additional information regarding a specific topic (his reemerging stuttering). No second interviews with the other participants were considered necessary.

### 2.4. Data Analysis

The interviews were transcribed verbatim by first author (EA) and the transcriptions were analyzed using qualitative content analysis [21]. During the following steps of the analysis, all three researchers were involved. Transcriptions and memos were read several times to get a sense of the whole. Subsequently, sentences and paragraphs were separated into meaning units which were condensed (shortened but with preservation of the content) and labeled with codes, by hand. In the next step of the analysis, the coded meaning units were compared across units of data, searching for similarities and differences. Thereafter, all the condensed meaning units were grouped into subthemes. A few subthemes were of subordinate nature in relation to the aim of the study and were sorted out as unrelated (such as descriptions of early experiences related to the onset of disease). After that, the subthemes were compared and related to each other and then merged into to following three overarching themes [22]: “increased and unexpected communication difficulties”, “strategies to improve communication”, and “mixed feelings surrounding the surgery”. The emergence of the theme “increased and unexpected communication difficulties” is described in Table 2. The themes and subthemes were regularly revised and refined, and definitions were further specified. Also, memos, subthemes, and themes were regularly discussed with the coauthors to strengthen trustworthiness. Quotations have been included in the findings section to illustrate and verify the interpretation.

## 3. Findings

All the participants described changes in different aspects of speech and communication as an effect of STN-DBS, both the surgery and the stimulation. These changes included a weak and monotonous voice and reduced speech intelligibility. However, the overall benefits of the surgery in terms of increased mobility were stressed by all participants. Despite different side effects, they still felt that they “had got their life back” as a result of the surgery. In addition, they were convinced that the progression of their disease symptoms had left them with no choice other than to have the surgery.

The content analysis resulted in 3 themes and 13 subthemes (Table 3). The themes are described in more details and exemplified with quotes below. 

### 3.1. Increased and Unexpected Communication Difficulties

All four participants reported varying degrees of negative effects on their speech after surgery. One participant did not have any speech or voice problems before the operation but developed difficulties after surgery. Others had dysarthria which worsened after the operation. Some symptoms increased, such as reduced intelligibility and problems with writing. 

In addition to a more or less expected deterioration in speech and communication, a number of unexpected difficulties related to communication were described. One participant reported the re-emergence of stuttering after surgery and another described a change in self-perception of her own speech, considered related to auditory feedback. All the participants reported an increase in mental fatigue and difficulty in concentrating for longer periods of time, which had an effect on their social life.

#### 3.1.1. Weak and Monotonous Voice

One participant mentioned that her speech improved post surgery, but gradually deteriorated again. The participants described their voices as weak, stiff, and monotonous. *“Yes, sort of whispering” (Sven). *The voice was less nuanced and rigid compared with before the surgery, which was commented on with sadness as a loss.* “The voice appears less nuanced to me, I cannot vary it like I did before. Without that, it gets stiff, that's sad.” (Lisa).*

#### 3.1.2. Vulnerable Speech Function Affects Intelligibility

Some participants experienced that other people frequently had difficulty hearing and comprehending their speech; their communication partners had to request clarification over and over again. *“No one hears what I am saying.” (Greta). *Moreover, the participants found that the speech difficulties became worse when they were tired. *“I do notice that it [the speech] is affected, by fatigue among other things.” (Sven). *Furthermore, the participants said that anxiety played an important role and reported reduced intelligibility when they were nervous or tense. *“It [the speech] should be better now, but it is not, it is being affected by nerves.” (Greta); “Yes, it [anxiety] has an impact, not just a little but a lot.” (Sven). *They also said that participating in the interview probably had a negative effect on their speech, since it made them nervous.

#### 3.1.3. Stuttering

One of the participants had stuttered as a child, which was reported to have disappeared at the age of 8–10 years. After surgery, the stuttering re-emerged and had been permanent ever since. *“No, it [the stuttering] appeared when they turned on the stimulation.” (Anders). *The stuttering consisted of frequent word-initial-syllable repetitions and blocked speech sounds, particularly on “good bye”, which he had difficulty pronouncing. The stuttering appeared in different situations but in all long conversations. If he concentrated on speaking slowly, he was easier to understand. This participant reported that his speech became better, with fewer instances of stuttering, when electrostimulation was reduced.

#### 3.1.4. Difficulty Reading and Writing

Writing appeared to have been micrographic for all participants before surgery, but the difficulties increased after surgery up to the point where the handwriting was not readable. None of the participants reported difficulty reading before the operation, but one of them reported reading difficulty after surgery. He described the reading impairment as similar to his writing difficulties, as the text merged and lagged behind and was difficult to focus on.* “Reading is much more difficult now compared with before the operation /…/ it merges somehow and lags behind.” (Sven).*

#### 3.1.5. Change in Auditory Feedback

One of the participants felt that the sound of her own voice sometimes changed, but this change was not perceived by people around her. She reported that her voice sounded as though she was talking in a bucket or a can. *“Sometimes I think I sound as if I was speaking in a can, my speech is becoming very hollow.” (Lisa).*

#### 3.1.6. Mental Fatigue

The participants reported not having the same social capacity after surgery. After a short time in a large group of people, they became tired. *“… but of course, we don't socialize with people as much as we did before.” (Lisa). *One of the participants mentioned that it had become more tiring to talk to friends over the phone, something she had enjoyed doing before the operation. She described it as an inability to listen for longer periods of time. *“I have lost patience when it comes to talking over the phone, I hear, but I don't have the strength in some odd way. /…/ I almost have to interrupt phone conversations if they are too lengthy.” (Lisa).*

#### 3.1.7. Freezing of the Mind

A couple of participants experienced word retrieval difficulties but also something they described as a “freezing of the mind”. It was not only a question of finding the right words but rather an inability to remember anything because the mind suddenly turned absolutely blank. *“My brain stops more than before. If I am going to say something, it can suddenly shut down and I have no chance of thinking of what I wanted to say.” (Lisa) *This fear of the mind becoming completely blank made the participants feel insecure and made them sometimes decide not to participate in a conversation, because of the risk of being unable to finish a story. The freezing of the mind was described in terms of insecurity and loss of control. *“It's like deciding whether to dare or not dare; if I get going, I'm not sure I will be able to end the story or whatever I am going to say.” (Lisa); “… there seems to be nothing to hold on to, I am just jumping from one thing to another.” (Sven)*

### 3.2. Strategies to Improve Communication

As a result of the different speech and communication problems experienced by the participants and described above, they had found ways to improve communication. They reported using different strategies, such as adjusting speech rate and loudness in order to increase speech intelligibility. It was reported that communication partners played an important role in solving communication problems in conversation. Furthermore, the participants said that the level of medication and stimulation parameters were of importance to their speech and communication.

#### 3.2.1. Speech Techniques

Most participants made conscious use of strategies such as adjusting speech rate. They tried to talk more slowly, as it made them more understandable. *“When I talk without focusing, I am difficult to understand.” (Anders); “[I] talk very slowly.” (Greta). *They were also aware of the importance of increasing vocal loudness to make their voices more powerful. One participant described adjusting her voice as being much like turning up the volume on a radio. If she did not, she would be asked to repeat herself again.* “It's as if I turn up the volume of a radio, to make other people satisfied. Otherwise they will ask me to repeat what I say.” (Lisa).* They also chose to adjust their social schedule to times where they were less tired or more intelligible. 

#### 3.2.2. Communicative Partners' Support

The participants described being difficult to understand in various situations which made participating in social life difficult. For some social functions, the ability to make phone calls is important, something that was considered particularly difficult by some of the participants. This made them dependent on other persons.

The need for several repetitions in order to be understood also led to increased dependence on family and relatives to help out in communicative situations. One participant stressed the importance of having family who knew her very well because when her mind “froze” and she did not remember anything, her husband would fill in or explain the situation to other communication partners. *“My husband is so wonderfully knowledgeable about my life, he can help me along or he lets me practice, because it has become better.” (Lisa). *All the participants described a changed balance in the communication situation, with the partner having to put more effort into the communication and paying more attention in order to keep conversation going. *“Then it's the other partner that has to be attentive and make an effort to get it [the conversation] going.” (Lisa) *This change in communication pattern had social consequences, described as loneliness and restricted social participation.* “Then it's the social part, you realize who your friends are now.” (Anders).*

#### 3.2.3. Changing Medical and Stimulation Parameters

After the operation, all four participants had been able to reduce their medication considerably, which was perceived as an important improvement. However, adjustments were necessary and varying effects of medication after surgery were reported.* “It is when I am under-medicated that it gets worse.” (Lisa). *One participant increased her medication to increase mobility, which made speech difficulties worse. *“If I take more medication, I can walk but not talk, it's just a matter of choice.” (Greta). *She could choose whether to prioritize walking or talking in a specific situation. Another participant had the opposite experience: increased medication improved speech function. Moreover, the effects of medication varied during the day. In overall terms, this variability made the participants adjust their daily schedule to optimize the performance of various activities.

Stimulation settings were also perceived as affecting speech and communication. In particular, one participant had the opportunity to adjust the stimulation parameters himself and made conscious choices to increase his ability to communicate versus moving around. *“When I am going to talk for a longer period of time, an hour or so, I can lower the stimulation, to be able to speak more easily. Afterwards, I increase it again to be able to move more easily.” (Anders).*

### 3.3. Mixed Feelings Surrounding the Surgery

All four participants described the decision to go ahead with the surgery as a dramatic and unavoidable one, because of increased disease symptoms and less levodopa effect. They all described feelings of uncertainty: they lacked information pre- and postsurgery and also wanted information about possible future changes. In spite of this, they were happy with the decision to have surgery because of the general improvement in quality of life, despite the perceived negative effects on speech and communication.

#### 3.3.1. An Unavoidable, Drastic Decision

The participants described their time presurgery as being in a very bad medical state, desperate for a change, willing to try almost anything. *“I was in such a bad state, I thought it could not get any worse. And that's when they take such drastic measures. Because that's what this operation still is, isn't it?” (Lisa). *They described increased severity of symptoms such as dyskinesia, freezing, bradykinesia, and tremor. *“I was ready for an operation when the rigidity and the dyskinesia superseded each other, so that I did not get any good time in between.” (Lisa). *Also out of concern for significant others, the decision to agree to the operation was described as unavoidable.* “I had to medicate every hour and so I had to have an operation.” (Anders).*

#### 3.3.2. Improved Quality of Life

All the participants expressed a feeling of being pleased that the operation was worth it, because of a general increase in quality of life. Although they all experienced adverse effects, they “got their lives back”. *“Everything got better, the tremor disappeared completely. It was fantastic to wake up.” (Greta).*

The two women did not express any disappointment at all, although they both experienced a worsening of speech symptoms. The increased mobility and reduced tremor made up for everything. *“I feel that I could not have managed without the operation, I have not regretted it for a single moment, because it meant so much to be able to move again.” (Lisa). *The two men described having higher expectations compared with the actual outcome and were therefore somewhat disappointed. However, the increased independence was acknowledged as a major improvement.* “I could not get up at night, D had to help me a lot at night /…/ now I can manage completely on my own.” (Anders).*

#### 3.3.3. Uncertainty and Lack of Information

Some of the participants felt that they needed more information, both before and after surgery, about what to expect in terms of possible side effects, and also how the disease could be expected to develop as a result of surgery. They expressed disappointment at the amount of information that was offered and one participant suggested meeting with other patients who were treated with STN-DBS. *“I am a bit disappointed that you are unable to get information /…/ they could have rounded up a few [operated patients] to give me tips on how it really is.” (Sven).*

The participants had different thoughts about the future. The disease symptoms changed constantly and the participants needed to deal with these changes continually. Feelings of uncertainty were described by all participants. They wanted to know how both the disease and the speech impairment would develop. *“All the time, when one thing stops, when the pain or rigidity or whatever it might be gets better, it's time for something new /…/ I would like to know, is this rigidity it, or does it get worse? What is going to be next?” (Sven). *They also described fear about starting new projects because they were uncertain whether they would be able to follow them through.* “I'm in a situation where I do not know if I dare to start something because I do not know if I will be able to finish it.” (Lisa).*

## 4. Discussion

To summarize, this study investigated how speech and communication were perceived by four individuals with Parkinson's disease, following surgical treatment with deep brain stimulation to the subthalamic nucleus. The participants described different improvements such as increased mobility and a radical reduction in tremor and medication. At the time of surgery, they all felt that they were in such a bad medical state that they had no other choice but to agree to the surgery. Furthermore, they said that speech and communication deteriorated as a result of surgery and/or stimulation.

### 4.1. Speech and Communication

The findings of the present study add an in-depth individual perspective to what is known about the speech effects of subthalamic stimulation. The reports from the participants described reduced speech intelligibility, which agrees very well with recent group studies (e.g., [3]), in which a significant reduction in speech intelligibility was reported. However, Tripoliti et al. [3] noted that there was a substantial individual variability. In their group of participants, speech intelligibility deteriorated in 25 patients (varying between −77% and −3%) and improved in 7 patients (ranging from 2% to 17%). Tripoliti et al. also reported that acoustic speech measures of vocal loudness increased in all speech tasks with stimulation, which can be related to other findings of speech subsystem improvements (e.g., [5, 6]) and can be explained by an STN-DBS-induced increase in force production but cause a deterioration in more complex movements. The four participants in the present study mentioned vocal weakness as a consistent problem. The self-report questionnaire, the Voice Handicap Index (VHI), was used to describe the perceived voice problems after surgery by Frost et al. [13], and 14 of 20 participants rated their perceived current voice difficulties greater than before surgery, but only VHI means and no particular perceived symptoms were reported in the study, which makes a comparison difficult.

One of the participants reported the re-emergence of developmental stuttering, not as a symptom of the disease *per se* but in connection with the surgery and also said that the severity of disfluencies was influenced by the intensity of stimulation. Emerging, reemerging, or increased disfluencies as an effect of STN-DBS are corroborated by clinical observations, but, as far as we know, they have rarely been described. Burghaus et al. [23] published a case study describing the aggravation of stuttering in a person treated with STN-DBS. On the other hand, Walker et al. [24] described the relief of stuttering symptoms in an individual with PD as an effect of unilateral STN-DBS. Clearly, there is a need for further studies exploring the role of the basal ganglia circuitry in the pathophysiology of disfluency. 

The description of mental fatigue, freezing of the mind, and vulnerable speech function is an illustration of the way cognitive factors and cognitive decline can be influential. This makes the contribution and support of significant others important. Communication partner support is important for individuals with dysarthria caused by Parkinson's disease in general [12, 25], but it might be even more crucial for patients treated with STN-DBS, a treatment which entails both increased and unexpected speech and communication changes.

### 4.2. Improved Quality of Life but Lack of Information

This study confirms numerous studies describing the dramatic positive effects of STN-DBS on mobility and tremor. None of the four participants regretted going through with the surgery, despite the fact that they experienced side effects. Bearing in mind the very small number of subjects, a possible gender difference in the perceptions of surgery outcome might exist. Apart from the fact that the proportion of male patients who undergo STN-DBS exceeds the reported male/female ratio of PD patients [26], gender-specific symptoms as a consequence of STN-DBS have been described. Like the participants in the present study, women frequently experience greater benefits in terms of perceived quality of life [27], although a recent study points to transient poorer outcome, not measurable at follow-up 3 and 5 years after surgery [28].

One of the factors that could increase patient satisfaction is clearly increased information relating to the procedure. The participants in the present study described a need for more information, regarding both the surgery and the possible side effects. This is consistent with the study by Montel and Bungener [29] in which a group of 40 STN-DBS treated patients were compared with a non-surgically treated matched group. The only differences between the groups in terms of quality of life and coping strategies were lower scores on the communication area of quality of life and also lower scores on instrumental coping strategies in the STN-DBS group. The authors stress the need to prepare patients with PD for the effects of surgery and stimulation and point out that the opportunity to meet other subjects who have already undergone stimulation could help the patient to develop a more realistic view of the intervention. 

### 4.3. Study Limitations and Directions for Future Research

Trustworthiness aspects are discussed in terms of credibility, confirmability, dependability, and transferability [30]. The collected data were handled according to the principles of content analysis, including continuous discussions between and the involvement of all the coauthors to increase credibility [31] and to reduce researcher bias. As speech and language pathologists, the researchers had a preunderstanding of speech and language problems which could have biased the study; a multiprofessional team could have further strengthened credibility. Investigator responsiveness, that is creativity, sensitivity, and insight, was also supported during the data collection phase, by conducting pilot interviews to refine interview skills for instance. Concerning confirmability, the researchers strived for openness and verification, by systematically checking and confirming the relationship between the data and the interpretation. To strengthen dependability, an interview guide was used in all the interviews. As for transferability, there was an attempt to present the analysis and the findings transparently and clearly, for instance, by providing illustrative quotes. The very small number of subjects is obviously a limitation of this study. More participants would have increased transferability and a larger interview or questionnaire study is clearly needed. There were also large differences between the participants regarding time after surgery, which might have influenced the findings. However, the interviews were carried out according to the procedures of qualitative research interviews with the aim of obtaining rich and consistent descriptions of the participants' experiences [32]. 

There are divergent meanings of using participant validation, therefore this was not done. A participant may have changed his/her views due to temporal aspects and other potential changes in his/her situation [21]. Moreover, a problem of participant validation is that the findings have been synthesized, decontextualized and abstracted from (and across) individual participants, so that an individual participant might not be able to recognize him/herself from the presentation of the findings [33]. 

### 4.4. Clinical Implications

It was stated in the introduction that the one consistent finding in studies of STN-DBS in PD is variability and the participants in the present study add to this variance. Because of the individual variability, as Montel and Bungener [29] pointed out, individuals with PD need to be informed in detail about the effects of surgery and of stimulation before the intervention. This is confirmed in the present study; therefore, the prospective patients need to be prepared and the preparation should be adapted to their specific symptoms, both general and in terms of speech and communication difficulties. Moreover, the information should be based on their expectations, and meeting other patients who have undergone the procedure would improve their understanding of the procedure. The development of a questionnaire, specifically tailored for this group of patients, would be helpful in identifying and formulating the concerns. Furthermore, voice and speech therapy, before or after surgery, focusing on skills and strategies to handle the changes in speech and communication, could facilitate the adjustment to the new speech conditions.

## Figures and Tables

**Table 1:** Demographic data (all names are pseudonyms).

Participant	Gender	Age years	Diagnosis	Disease duration (years)	Years after STN surgery	Total score UPDRS before STN surgery	Total score UPDRS after STN surgery (12 months )	Speech before surgery	Speech after surgery (6–12 months )	Speech intelligibility before STN surgery	Speech intelligibility *** after STN surgery
Lisa	Female	71	Idiopathic Parkinson's disease	24	4	Without L- dopa: 37 With L-dopa: 9	Med + stim + 21 Med − stim + 21 Med – stim – 30	No dysarthria Dysarthria assessment mean score 0.1**	No dysarthria Dysarthria assessment mean score 0.03	Words: 100% Sentences: 100%	Words: 100% Sentences: 98%
Greta	Female	73	Idiopathic Parkinson's disease	32	10	No data found	Med + stim +10 Med – stim + 14 Med – stim − 23	Mild* hypokinetic dysarthria Dysarthria assessment mean score 0.49	Mild hypokinetic dysarthria Dysarthria assessment mean score 0.92	Words: 96% Sentences: 94%	Words: 96% Sentences: 95%
Anders	Male	61	Left sided hemiparkinsonism	10	2	Without L-dopa: 30 With L-dopa: 62	Med + stim + 26 Med – stim + 35 Med – stim − 50	Mild-moderate mixed dysarthria, speech festinations Dysarthria assessment no data found	Dysarthria Dysarthria assessment mean score 1.0	Words: 70% Sentences: 60%	Reading**** 95% Spontaneous speech: 85%
Sven	Male	79	Idiopathic Parkinson's disease	30	9	No data found	No data found	Mild hypokinetic dysarthria Dysarthria assessment mean score 0.36	Mild hypokinetic dysarthria Dysarthria assessment no data found	Words: 99% Sentences: 100%	No data found

*Dysarthria classification: mild = speech affected but intelligibility intact, moderate = intelligibility slightly decreased, severe = speech supported by augmentative and alternative communication [18].

**Clinical dysarthria test, range 0–4 (normal <0,25; 4 = severe disability) [19].

***SWINT-Swedish Intelligibility Test, [20]. ****No formal SWINT judgment, speech intelligibility was rated in reading and spontaneous speech.

**Table 2:** The emergence of the theme “increased and unexpected communication difficulties”.

Quote	Code	Sub-theme	Theme
*“The voice appears less nuanced to me, I cannot vary it like I did before. Without that, it gets stiff, that's sad.” *	Voice less nuanced and stiff, that is sad	Weak and monotonous voice	Increased and unexpected communication difficulties
*“My brain stops more than before. If I am going to say something, it can suddenly shut down and I have no chance of thinking of what I wanted to say.”*	Mind suddenly stops in the middle of communication	Freezing of the mind	

**Table 3:** Overview of findings.

Themes	Subthemes
(1) Increased and unexpected communication difficulties	(1.1) Weak and monotonous voice
	(1.2) Vulnerable speech function affects intelligibility
	(1.3) Stuttering
	(1.4) Difficulty reading and writing
	(1.5) Changed auditory feedback
	(1.6) Mental fatigue
	(1.7) Freezing of the mind

(2) Strategies to improve communication	(2.1) Speech techniques
	(2.2) Communicative partners' support
	(2.3) Changing medical and stimulation parameters

(3) Mixed feelings surrounding the surgery	(3.1) An unavoidable, drastic decision
	(3.2) Improved quality of life
	(3.3) Uncertainty and lack of information
